# Cooperative social clusters are not destroyed by dispersal in a ciliate

**DOI:** 10.1186/1471-2148-9-251

**Published:** 2009-10-14

**Authors:** Nicolas Schtickzelle, Else J Fjerdingstad, Alexis Chaine, Jean Clobert

**Affiliations:** 1 Biodiversity Research Centre, Université catholique de Louvain, Croix du Sud 4, 1348 Louvain-la-Neuve, Belgium; 2 Department of Biology, Queens College, City University of New York, Flushing, NY, USA; 3 Station d'Ecologie Expérimentale du CNRS à Moulis USR2936, 09200 Saint-Girons, France

## Abstract

**Background:**

The evolution of social cooperation is favored by aggregative behavior to facilitate stable social structure and proximity among kin. High dispersal rates reduce group stability and kin cohesion, so it is generally assumed that there is a fundamental trade-off between cooperation and dispersal. However, empirical tests of this relationship are rare. We tested this assumption experimentally using ten genetically isolated strains of a ciliate, *Tetrahymena thermophila*.

**Results:**

The propensity for social aggregation was greater in strains with reduced cell quality and lower growth performance. While we found a trade-off between costly aggregation and local dispersal in phenotypic analyses, aggregative strains showed a dispersal polymorphism by producing either highly sedentary or long-distance dispersive cells, in contrast to less aggregative strains whose cells were monomorphic local dispersers.

**Conclusion:**

High dispersal among aggregative strains may not destroy group stability in *T. thermophila *because the dispersal polymorphism allows social strains to more readily escape kin groups than less aggregative strains, yet still benefit from stable group membership among sedentary morphs. Such dispersal polymorphisms should be common in other social organisms, serving to alter the nature of the negative impact of dispersal on social evolution.

## Background

Aggregative and dispersive behaviors could be antagonistic in many systems since high mobility should reduce the formation of aggregative associations. If aggregative behavior confers substantial benefits (e.g. via cooperation among kin), high dispersal destroys these benefits by reducing group stability [1]. Under these conditions, one should expect social species to have much lower dispersal than solitary species producing an aggregation-dispersal trade-off. The connection between dispersal and aggregation or cooperation, however, is far from straightforward. In a characteristically groundbreaking discussion of the issue, Hamilton and May [1] suggested that while aggregation among kin can indeed drive kin cooperation, as aggregations grow so does competition among related individuals (kin competition). Such strong competition among kin hence engenders inclusive fitness costs of aggregation that could be ameliorated by dispersal [2,3], but dispersal reduces group stability, and stability should promote cooperation [4-6]. Because aggregation can lead to more advanced forms of cooperation, including altruism [7-10], understanding the conditions that allow for increased aggregation despite costs associated with this behavior are of intense interest to evolutionary biologists [10,11].

At the extreme, a trade-off between aggregation and dispersal is expected as described above, and a negative relationship between these two parameters has been a basic assumption of many models examining the evolution of social behavior [11-14]. Some theoretical studies have shown that high dispersal and cooperative behavior are incompatible when populations are saturated [4,15-17]. However, very low levels of dispersal lead to competition that occurs primarily among kin, which can exactly cancel the benefits of cooperative behavior [16]. A small amount of dispersal is always necessary for cooperative strategies to spread in a population and so that the burden of increased competition is not exclusively among kin. Hence, while a trade-off between dispersal and cooperation is expected, the nature of this relationship shows extensive variation among different theoretical formulations and the coexistence of dispersal and cooperation will likely depend on life history assumptions.

A few theoretical studies have examined the coevolution of dispersal and cooperation with both parameters allowed to vary and have found that the spatial scale of competition and cooperation relative to dispersal distance is a critical factor governing the nature of a trade-off between dispersal and cooperation [15,18-21], reviewed in [13]). In essence, cooperators must disperse far enough to leave the kin group to avoid kin competition at high densities [19,21]. For example, more cooperative groups might produce individuals that are highly sedentary to take advantage of kin structure and long distance dispersers who colonize new habitats with no kin-competition (e.g. [22,23]). In general, then, resolution of the conflict between cooperation and dispersal occurs either through the disappearance of cooperation [24] or through specific behaviors that allow cooperators to maintain group structure despite dispersal (budding: [17], founding events: [8], social clusters: [25-27], temporal separation of cooperation [9,11]).

Empirical studies have shown that dispersal occurs even in the most social species, suggesting that specific behaviors that mediate the relationship between cooperation and dispersal may be widespread. For example, many social hymenoptera (e.g. ants) produce mating swarms among alates that end in the founding of new colonies considerable distances from natal nests [8,28]. The founding of a new nest by a single queen allows for high levels of relatedness while still reducing competition among sisters after dispersal. A few empirical studies have also explicitly investigated variation in the link between dispersal and aggregative or cooperative behaviors within a population [24,26]. For example, in *Pseudomonas aeruginosa *bacteria, reducing kinship or increasing kin-competition - both behaviors related to increased dispersal in wild systems - destroyed the social cooperation seen in this species [24]. However, in a more natural context, such bacteria are thought to reduce competition while preserving kin cooperation by budding off small social groups into new underutilized habitat [17]. In side-blotched lizards, *Uta stansburiana*, strong kin competition favors high juvenile dispersal, and cooperation among adults is only possible through greenbeard recognition of genetically similar individuals [26]. The variation in dispersal-aggregation trade-offs among species [29] could be due to a broad range of specific strategies used to mediate the inherent conflict between aggregation and dispersal and modulate the nature of this trade-off.

**Figure 1:**
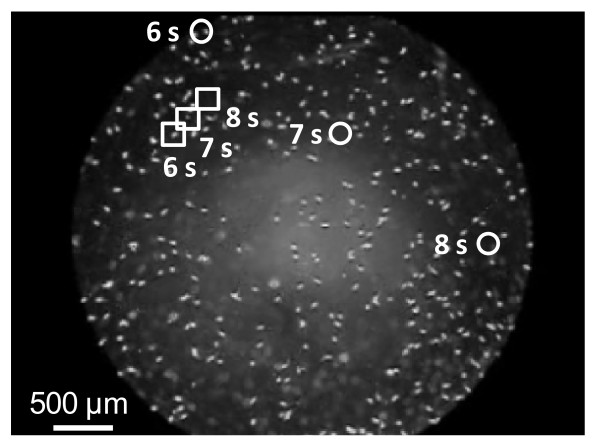
***Tetrahymena thermophila *dispersal morphs move substantially faster than normal morphs**. Shown are the positions of two *T. thermophila *cells at three successive time points (6, 7 and 8 seconds from start in the movie accompanying our previous paper [30]; picture background is for time = 6 seconds), illustrating the much higher swim speed and net displacement of elongated dispersal morphs (circles) compared to round sedentary morphs (squares).

We investigated the link between cooperative behavior and dispersal in experimental microcosms of a unicellular ciliate, *Tetrahymena thermophila*, to understand the potential and nature of trade-offs between these traits and to detect the consequences of this relationship on other life history characteristics. In this species, dispersal occurs naturally and is ecologically relevant since individuals occupy patchy ephemeral feeding habitats (F.P. Doerder, pers. comm.). Our previous work with *T. thermophila *showed considerable variation in the propensity to disperse among ten genetically isolated strains and this variation is linked to many other life history traits [30]. Also, our work and that of others [31] has shown that cell morphology is linked to dispersal and movement in this species: highly elongated cells (dispersal morphs) are capable of long distance dispersal due to growth of flagella [31], somewhat elongated cells are capable of short distance dispersal, and round cells are fairly sedentary (although not immobile; Figure 1). In addition, aggregation in this species, which occurs more readily among kin (unpublished data), entails cooperative behavior through the exchange of growth factors that improve cell survival and growth at low density [32], making the balance between aggregation, dispersal, and colonization a key feature of their evolutionary history. In this context, we tested 1) whether there was variation in aggregative behavior among ten genetic strains, 2) if a dispersal-aggregation trade-off existed among strains, and 3) if associations between aggregation and other core life history traits (measured in [30]) provided evidence for dispersal-aggregation syndromes within this species.

## Methods

### Strains, cultures, experimental design and data collection

This study is based on measures of core life-history traits of ten strains of *T. thermophila *gathered through five different experiments. The first four experiments have been described in detail in a previous study [30], which also gives basic information on strains, culture conditions, general manipulation of *T. thermophila *and data collection using digital imaging. Here, we summarize the main elements of the four published experiments, and then present the fifth in full details.

The first experiment estimated growth rates of strains in the presence of nutrients by initializing populations at low density (10000 cells/ml) and taking population density counts over eight days. Each cell line had three replicates for this population growth experiment. The second experiment assessed survival under starvation by placing cells in a nutrient-absent media and measuring decreases in cell density over 17 days. Starvation survival was estimated through three replicates per cell line. The third experiment evaluated dispersal rates of strains by introducing cells into one side of a two-patch experimental system (two 1.5 ml tubes linked by 17 mm long tubing) and measuring dispersal to the second patch over 17 hours. Dispersal was assessed through six replicates per cell line (exceptions due to technical problems: five replicates for strains 4A and Q, and three for strain B). Finally, the fourth experiment studied the capacity of strains to colonize a new patch without cell-cell cooperation by seeding new populations with a single cell and estimating survival and cell division rates after eight days. Ten cells were isolated for each cell line to determine the percent survival for each cell line from the percentage of coolies found among the ten tubes, and the whole experiment was replicated twice, giving two replicates per strain (except strain E where one replicate failed).

**Figure 2:**
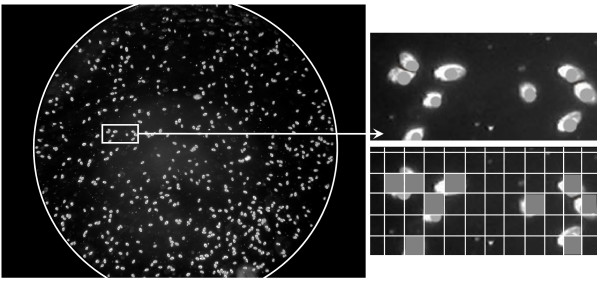
**Example of a digital picture of *Tetrahymena thermophila *used to quantify aggregation behavior**. The circle indicates limits of the study area, corresponding to the viewing field through the microscope. The magnified portion illustrates the point location (grey dots) of cells as computed by ImageJ analysis software, and the grid approximation used to compute the point pattern statistics via the Programita software (see text for details).

A fifth experiment, measuring the aggregation behavior of strains, is reported in the present study. Due to constraints on manipulation time, this experiment was performed in an incomplete block design, with five strains per day randomly allocated to each of four experiment days. Thus each strain was studied two times and originated from mother cultures separated by a number of generations (as in [30]). On each experimental day, five replicate samples were isolated into separate 1.5 ml Eppendorf tubes from the source culture of each strain. Culture tubes were homogenized through gentle vortexing, and appropriate volumes of cells were transferred into the five replicate tubes in order to create a density of 300 000 cells/ml in tubes with 500 μl medium. Tubes were left for one hour at room temperature for cells to acclimate to their test density. Then, one 10 μl aliquot was taken out from each of the five tubes, again homogenized through gentle vortexing, and loaded onto a count chamber of a Plexiglas slide (Hycor Glasstic slide with ten cell count chambers) placed under a microscope. Cells were then allowed to aggregate at will over 20 minutes inside the cell count chambers before a digital picture was taken of each count chamber (Figure 2). The experimental design therefore led to the measurement of aggregation for two repetitions of five replicate tubes each for each of the ten *T. thermophila *strains, giving ten replicates per strain (exceptions due to technical problems: five for strain B and nine for strain D2).

### Quantification of the degree of cell aggregation

Pictures were analyzed with ImageJ software (v. 1.40 g, National Institutes of Health, ; an updated version of the Scion Image used in [30]) to extract descriptors of the cells, including their position in an X-Y coordinate system. The aggregation tendency of cells on each picture was assessed with spatial point pattern statistical analyses using Programita software [33]. We created SAS [34] codes to automatically make Programita input files, read Programita result files, and conduct statistical analyses on these aggregation data.

Point pattern analysis is used by biologists to infer the existence of underlying spatial processes, like cell aggregation. A large number of methods have been developed but are all largely based on the same idea [33]. First-order statistics describe the intensity λ of points and its large-scale variation. Second-order characteristics are summary statistics based on the distribution of all point-to-point distances, and are used to detect different types of patterns and their associated scale via the quantification of small-scale spatial correlation structure of the point pattern.

We quantified the degree of aggregation of the point pattern (i.e. the cells present on a given picture of a cell count chamber) by the univariate pair-correlation function g(d), which gives the expected number of points at distance d (called scale) from an arbitrary point, divided by the intensity λ of the pattern. Aggregation is indicated by g(d) > 1 (there is a higher density of points than expected), whereas g(d) <1 indicates regularity of the pattern at distance d (there is a lower density of points than expected). Contrary to the classical Ripley's K or L functions, g(d) uses rings instead of circles, and is therefore not a cumulative function [33]. We used the grid-based numerical approach implemented in Programita to estimate g(d), because this method prevents biases due to edge effects regardless of the study area shape [33]; in our case this shape corresponds to the approximately circular viewing field through the microscope (Figure 2). The following parameter values were used in Programita for all point pattern analyses: grid size = 22.72 pixels, corresponding roughly to the average *T. thermophila *cell size (~50 μm; Figure 2) (this gives a grid of 100*75 cells for our 2272*1704 pixel digital pictures); scale d = 0 to 37 cells (i.e. up to half the lowest dimension, as recommended maximum: [33]); several points per cell allowed; ring width = 2.

**Figure 3:**
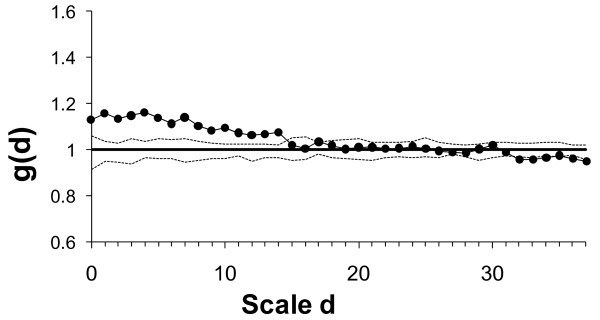
**Quantification of cell aggregation from point pattern analysis**. The pair-correlation function g(d) gives the expected number of points at distance d from an arbitrary point, divided by the intensity λ of the pattern. g(d) > 1 indicates aggregation, g(d) <1 regularity of the pattern at distance d. In this example of the picture displayed in Figure 2, cells are aggregated up to a distance of 14. Dashed lines: 95% confidence envelopes obtained from 19 simulations.

The basic result for each point pattern (i.e. each picture) consists in the value of g(d) for d = 0 to 37 (Figure 3), from which we computed a single summary measure of pattern aggregation, which we denote "aggregation index g": the mean of g(d) values superior to 1 (indicating aggregation) on d = 0 to 15. We restricted the mean to include a maximum scale of d = 15 as we were interested in quantifying small scale aggregation and not large scale variations in densities. However, sensitivity analyses (not shown) suggest that this specific value of d = 15 to compute the mean g had little effect on the statistical significance and correlation patterns between aggregation index g and life history traits; this was also the case for the value of ring width = 2.

Correct positioning of contiguous cells, which are not distinguished in image analysis software, is a prerequisite for valid estimation of aggregation. Available image processing procedures (e.g. watershed separation) were not reliable at splitting partially overlapping cells in our pictures. We therefore statistically split groups of contiguous cells based on their size relative to the median size of all cells in the picture. The classes used (<1.6*median = 1 cell, 1.6*median to 2.6*median = 2 cells in a line, 2.6*median to 3.6*median = 3 cells placed as a triangle, 3.6*median to 4.6*median = 4 cells placed as a rectangle) were optimized to minimize the error rate (under- or over- splitting) by manually checking 3781 cell clusters (72% were correctly split, and errors were symmetrically distributed between under- and over-splitting). We also checked the validity of this procedure by comparing the aggregation estimate for each picture with this procedure and with a manual cell separation achieved by drawing black lines between contiguous cells on the pictures themselves. The correlation between the g value obtained by the two methods on the same picture was high (Pearson r = 0.86, n = 94, p < 0.001). Statistical splitting also has the advantages of greater efficiency and more reliable criteria for dividing partially overlapping cell clusters.

### Statistical analyses

Statistical tests for aggregation or regularity are usually based on the comparison of the observed g(d) with confidence envelopes obtained from simulations. Generally, this approach is subject to several problems [35] including the selection of an appropriate null model, but these were not relevant here because we were not interested in hypothesis testing (i.e. assessing the statistical significance) of the degree of individual point pattern aggregation. Our goal was to quantify the magnitude of cell aggregation, compare levels among strains, and measure correlations with other life history traits previously measured for *T. thermophila *[30]. A few preliminary tests on the aggregation index g allowed verifying that no artifacts or biases due to the incomplete block experimental design (four different experimental dates to obtain two repetitions per strain) or to the natural variation in cell density (λ) between pictures confounded our analyses.

Differences in cell aggregation between strains were studied by generalized linear models and discriminant analyses. The covariation of aggregation with other life-history traits was examined through Spearman's correlation and principal component analysis, all implemented using SAS software.

Despite clonal reproduction of our source cultures, variation among replicates is expected from three sources. First, each replicate will have small differences in density due to the estimation technique used to establish replicate populations and phenotypic traits could be in part influenced by external conditions among clones ([30], unpublished data). Second, the genetic architecture of *T. thermophila*, which includes both a germinal micronucleus and a macronucleus transcribed to produce the phenotype [36], lends itself to extensive phenotypic variation among clones. While the germinal micronucleus is faithfully copied during clonal fission, the macronucleus is reconstituted upon division and is usually composed of a different number of copies of a different subset of the micronucleus genes among daughter cells [36,37]. Finally, large population sizes, rapid generation times, and the additional transcription step in creation of the macronucleus will have introduced a significant number of mutations with the potential to yield random among-replicate variation during establishment of replicates.

For the above reasons, the between- and within strain variation in life history traits are both important to assess the existence of associations between life-history traits, aggregation, and dispersal among strains, even if this measure includes both genetic and non-genetic effects (plasticity, epigenetics including macronucleus sampling). Statistical analyses were therefore done on measures obtained at the replicate level. However, this approach could increase the risk of artificially augmenting the statistical power by increasing sample size (replicates within a strain vs. strain averages). Therefore, results are also given for the same analyses done at the strain level by averaging measures across replicates. The results from the two analytic methods are generally similar in magnitude and direction, but we have kept interpretation and discussion of the results more tentative in cases where statistical significance differed.

We conducted a number of independent experiments to measure each life history trait since it is not technically possible to measure all variables with a single experimental design. In addition, due to different time constraints faced by each protocol, some experiments had a different number of replicates. Since experiments were independent of each other, specific replicates of a given strain were not directly linked between all of the experiments. Therefore, in analyses that account for within-strain variation, we used a randomization procedure [38] to correlate parameters at the replicate level from different experiments, similar to the method used by [30].

## Results

### Aggregation among cell strains

**Figure 4:**
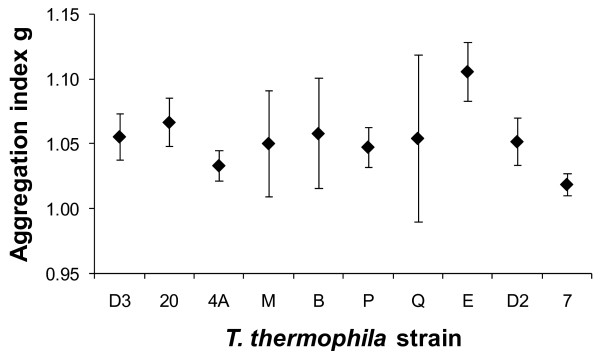
**Aggregation tendency of the ten studied strains of *Tetrahymena thermophila***. Mean (and 95% confidence interval) aggregation index g is reported, based on ten replicates per strain (exceptions due to technical problems: 5 replicates for strain B and 9 replicates for strain D2).

Aggregation tendencies of *T. thermophila *cells in our experiments, quantified as the aggregation index g, differed significantly among the ten studied strains with no significant variation between the two repetitions per strain (two-way incomplete block factorial design: strain: F_9,83 _= 3.03, P = 0.004, Figure 4; repetition: F_1,83 _= 0.21, P = 0.649). The degree of cell aggregation g was not significantly correlated to the mean number of cells captured in pictures, estimated by the mean point intensity λ (Spearman's correlation coefficient r = -0.140, n = 94, P = 0.179) as expected given its definition [33], though λ differed significantly between experimental dates (F_3,90 _= 11.74, P < 0.0001). Among-strain differences in cell aggregation were therefore not an artifact caused by capturing different numbers of cells in the pictures taken of cell count chambers. Furthermore, the aggregation index g did not differ significantly between dates (F_3,90 _= 0.28, P = 0.838).

### Aggregation-dispersal trade-off

**Figure 5:**
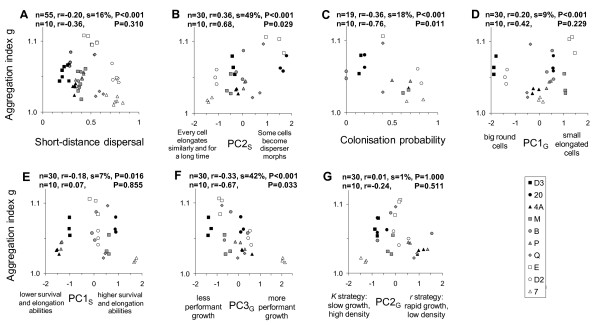
**Correlation of aggregation with seven other life-history traits of *Tetrahymena thermophila***. Those named "PC" are combinations of traits obtained from Principal Component Analyses; we describe their essence here but full details are given in [30]. The degree of cell aggregation under food rich conditions showed a negative relationship with short-distance dispersal including variation within and among strains, but not in more restrictive among strain analysis (A). Elongation strategy under starvation conditions was markedly associated with the tendency to aggregate, with strains where some cells elongated far more than others (up to becoming dispersal morphs) and for a shorter time showing stronger aggregation than strains where all cells elongated similarly for a long time (B). Strains that tended to aggregate strongly were less efficient as single-cell colonizers (C). Strains with small and elongated cells under food rich growth conditions showed a higher tendency to aggregate than strains with big and round cells, this effect was only present when within-strain variation was included (D). More aggregative strains showed reduced survival and average elongation abilities under starvation conditions when within strain variation was included in analyses (E). Strains growing faster and reaching a higher final cell density in the presence of nutrients were less inclined to aggregate (F). Growth strategy (*K *vs *r*) showed no relationship with aggregation (G). Because replicates of a given strain were not linked between experiments, we used a randomization procedure [38] to correlate parameters from different experiments, similar to the one used by [30]: the replicates of a given strain were randomly associated across experiments 1000 times, and a correlation was computed for each random association. n: sample size (limited by the experiment with the smaller sample size); r: mean Spearman's correlation over the 1000 random associations; s: proportion of significant correlations over the 1000 random associations; P: probability of obtaining s if the null hypothesis of no correlation is true. Points on each graph reflect the means of five random associations between the two traits to illustrate both between and within strain variations. The second line of statistics at the top of each graph gives results for Spearman correlations based on means of the 10 strains only, discarding variation between replicates of each strain.

More aggregative strains showed reduced short distance dispersal rates under food rich conditions (Figure 5A), although the strength of this relationship was fairly weak and only showed statistical significance when accounting for within-strain variation among replicates. The strength of the dispersal-aggregation relationship may have been weakened by specific dispersal strategies. Strains with strongly aggregating cells were characterized by an elongation strategy under starvation conditions where some cells elongated more than others, to the point of becoming dispersal morphs (vs a strategy where all cells elongate similarly for a long time: Figure 5B). That is, strains with a strong aggregative behavior also produced more of the morphologically specialized, rapid-swimming dispersal morphs.

### Associations between aggregation and life history traits

Differences among strains in the aggregation index g were significantly correlated to variation in other life history traits: cells from strains showing high levels of cell aggregation were poor at colonizing a new patch in the absence of clone mates and conspecifics (Figure 5C) suggesting a strong dependence on sociality. Associations also existed between aggregation and traits associated with cell growth. Aggregative strains exhibited a small and elongated cell shape under normal growth conditions (Figure 5D), survived somewhat less well under starvation conditions (Figure 5E), and had relatively poor population growth performance (Figure 5F) with food present, although the first two patterns were only significant when within-strain variation was taken into account. No relationship was found between aggregation levels and the cell line's growth strategy (*K *vs *r*: Figure 5G).

**Figure 6:**
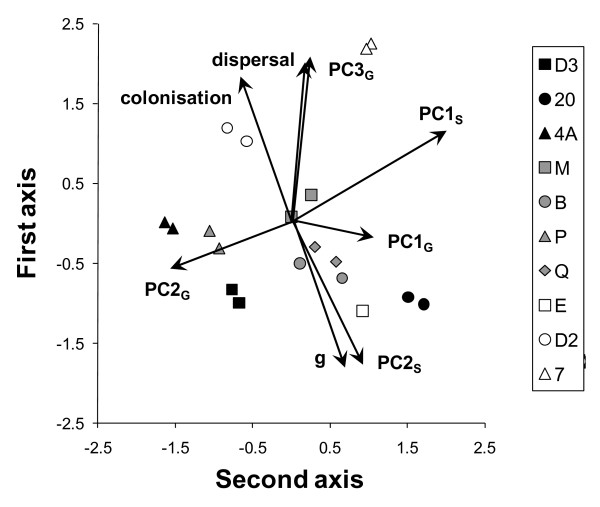
**Summary of the associations between aggregation and the seven other life-history traits of *Tetrahymena thermophila***. Principal component plot representing the associations between the first two component axes and component loading vectors for cell aggregation index g and the seven other life-history traits quantified for the ten *T. thermophila *strains studied [30]. Vectors that share a similar direction and length suggest traits that are more highly associated among cell strains. For the same reason that replicates of a given strain were not linked between experiments, points on the graph show means of five random associations between replicates of each strain to illustrate both between and within strain variation as in Figure 5. Results on analyses of the 10 strain means were extremely similar and are not shown.

The above associations, with strongly aggregating strains being poor single-cell colonizers with small cell size and poor growth performance in nutrient rich conditions, and a high production of long-distance dispersal morphs under starvation, were well represented by the first two axes of a principal component analysis including all the life history traits studied here (Figure 6). The first axis of this comprehensive PCA explained on average 36% of the variance (SD: 1.9%; range: 32% to 38%, over 1000 random associations of replicates across experiments; 43% when analysis was done on the 10 strain means) and the second axis 22% of the variance (SD: 1.6%; range: 20% to 25%; 23% when analysis was done on the 10 strain means). These two axes allowed us to accurately discriminate among strains, with only on average 22% (SD: 11.5%; minimum, median, maximum: 5%, 26%, 37%, respectively) of the replicates not correctly classified suggesting distinct life history strategies of each cell line. This latter analysis cannot be done if working on the 10 strain means only.

## Discussion

Our results show a trade-off between aggregation and dispersal (Figure 5A) in *T. thermophila *as expected at a very basic theoretical level, but this trend was only significant when taking into account within-strain variation. The difference between results from the two analyses suggests that this trade-off is fairly weak and not necessarily due to additive genetic variation. Given this pattern at the phenotypic level, increased dispersal should reduce group stability and thus the benefits of maintaining aggregative behavior. We also found that aggregative strains showed a reduced capacity for colonizing new patches from single cells (Figure 5C), which may reflect a reduced capacity to exchange growth and survival factors essential at low density [32]. This reduced colonization ability of more aggregative cells would produce strong selection against dispersal in aggregative strains and one would predict a strong negative association between aggregative and dispersive behavior among strains. However, the strength of the trade-off between dispersal and aggregation we detected in *T. thermophila *was not as strong as one might expect if aggregation reflects cooperation in this species and may exemplify the notion that dispersal-cooperation relationships could be far from straightforward [11,13,20].

Aggregative behavior is an essential component for cooperation to evolve since it allows individuals the proximity necessary to transfer information or resources. Such behavior has been documented in many taxa, ranging from *Pseudomonas aeruginosa *bacteria [24] to side-blotched lizards *Uta stansburiana *[26], and is characterized by both benefits and costs of social interactions. Aggregation in *T. thermophila *reflects cooperation through both the exchange in growth and survival factors at low population density [32] and longer population persistence among kin in more aggregative cell strains (unpublished data). In the present paper we also detected a number of potential costs associated with this cooperative aggregation behavior that were more prominent in analyses including within-strain variation. More aggregative *T. thermophila *strains had lower growth rates (Figure 5F) and showed a tendency to have smaller elongated cell shapes (Figure 5D) that could reflect lower resource acquisition and a reduced ability to divide. This lower resource base may have also contributed to the reduced survival rates of these clones when nutrients were suddenly removed (Figure 5E) found at the phenotypic level but not in analyses on averages across replicates. Increased dispersal would cause a loss in kin-based cooperative behavior (unpublished data) which counter-balances the phenotypic costs of aggregation as social structure becomes less stable and thus would lead to selection for reduced dispersal among more aggregative strains. Increased dispersal would make social structure less stable and so cause a loss in the kin-based cooperative behavior (unpublished data) that counter-balances phenotypic costs of aggregation, and this loss would lead to selection for reduced dispersal among more aggregative strains.

Two lines of logic lead to the expectation of a strong trade-off between dispersal and aggregation: 1) that dispersal will break apart aggregations and thus lead to the loss of cooperative group benefits, and 2) that high dispersal rates preclude the ability to form stable social groups necessary to evolve cooperation [4,29,39]. We detected a dispersal-aggregation trade-off consistent with basic theory in analyses including within-strain variation, but this relationship disappeared in analyses focused only on among strain variation suggesting the relationship is weak or due to non-genetic effects. The nature of this trade-off could be weaker than expected from theory due to specific adaptations that would allow cooperative aggregators to benefit from dispersal. For example if there is strong selection for dispersal (e.g. due to high levels of kin competition: [1] or habitat instability: [40]; reviewed in [39]), then both social and asocial strains should disperse and behaviors that allow social strains to find or create social groups after dispersal would be favored.

Kin-recognition (and/or greenbeard recognition) is one such adaptation that would allow strains to gain the benefits of dispersal and subsequently regroup among genetically similar individuals to benefit from cooperation. For example, in both ascidians and side-blotched lizards, kin or greenbeard-recognition allows genetically similar individuals to regroup and cooperate after juvenile dispersal [26,41]. Indeed, *T. thermophila *shows kin-recognition and kin-recruitment among cooperative strains which can both protect cooperators from invasion by cheaters as well as encourage cooperation among genetically similar individuals after a dispersal event (unpublished data). Thus kin-recognition in this species facilitates the evolution of dispersal among cooperators and could contribute to a reduction in the strength of a dispersal-aggregation trade-off that we found.

Context-dependent and phenotype dependent dispersal [42-44] provides a second mechanism that can alter the strength and nature of the relationship between dispersal and stable social group structure and could lead to more similar dispersal rates for social and asocial phenotypes. Dispersal to new patches will result in a loss of cooperative opportunities for social strains, endangering their survival and reproductive success, because they are likely to arrive in patches that are empty or occupied by other, potentially non-cooperative, strains (field samples suggest some mixing of strains in nature; [45], F.P. Doerder, pers. comm.). However, this cost could be ameliorated by phenotype dependent dispersal, if such dispersing morphs of aggregative strains are equipped for greater independence and can more easily start up a new colony or avoid competition, similar to cases such as highly social naked mole rats and many ants [8,46,47]. Consistent with this, we found that more aggregative *T*. *thermophila *strains showed a much greater variation in the degree of elongation among cells, producing some highly elongated cells and some very round cells (Figure 5B). Because elongation of this type can lead to long distance dispersal [30], aggregative strains appear to be making some highly dispersive individuals and some very sedentary individuals. In contrast, less aggregative strains show much less variation in cell morphology and less cell elongation suggesting that individual cells are more similar in their dispersive capabilities and travel shorter distances than 'cooperative disperser morphs'. Dispersal phenotypes may be a common solution to minimize the loss of cooperation during dispersal and colonization in highly social species.

The effects of kin-recognition coupled with a dispersal polymorphism provide important mechanisms to allow the benefits of dispersal without completely sacrificing the benefits of cooperation and stable group structure. Kin-recognition is used to modulate cooperative behavior and orient dispersal in this species (unpublished data), which would allow disperser morphs among cooperative strains to leave high density or over-exploited patches while still benefiting from cooperation upon arrival. Kin-oriented dispersal among cooperative strains has only been tested at shorter distances (unpublished data), so it remains possible that the highly elongated disperser morphs we detected among cooperators specifically target uninhabited patches. Indeed, one advantage of dispersal is to escape kin competition. Since cooperative strains suffer reduced colonization success, we might predict that disperser morphs among cooperative strains will have much higher colonization success as single cells than the average value we detected here from randomly selected individuals in the population. Alternatively, dispersal by *T. thermophila *'disperser morphs' might occur in groups [17] which would provide the benefits of cooperation at low population size [32], thereby facilitating the colonization of new habitat patches.

Production of alternative life history strategies detected in *T. thermophila *(here and [30]) stems from the integration of many different traits (Figure 5). We found extensive variation in the degree of aggregation among *T. thermophila *strains (genetically isolated cell strains) which was consistent across replicates, suggesting considerable genetic variation for aggregative behaviors. Variation in aggregative behaviors detected here is linked to variation in both the costs (this paper) and benefits (unpublished data) of cooperative behavior as well as dispersal ability [30] and a whole suite of other life history traits (here and [30]). A consequence of these linkages between traits is the trade-off between dispersal and aggregation, which in turn favors adaptations for dispersal among cooperative strains. The successful production of cooperative dispersal morphs most likely requires the coordination and integration of a large number of traits which might be greatly facilitated by outcrossing among specific combinations of 'mating types' [36]. Similar mating systems appear to be common among other single celled eukaryotes that show cooperative behaviors yet inhabit patchy and ephemeral habitats that favor dispersal [48-50] and may suggest that dispersal polymorphisms also exist in species such as *Dictyostelium *slime molds.

An emerging pattern in highly cooperative taxa (e.g. mole rats, aphids, ants, and *Tetrahymena*) appears to be that dispersal is characterized by specialized phenotype-dependent dispersers (see also [44]). When dispersal is favored [1,40], sedentary cooperative individuals will benefit from being able to take on a very different phenotype associated with higher mobility and the ability to exist in solitary conditions in order to successfully disperse. Consistent with this, dispersal morphs among cooperators would be capable of much longer range dispersal than non-cooperative individuals in *T. thermophila *since longer shaped cells are capable of longer distance dispersal [31]. Dispersal distance depends on many factors with cooperation being key [2,14,19]. Many highly social species have large group size (e.g. ants, bees, ground squirrels; [51]) thus requiring long distance dispersal to escape kin neighborhoods and kin competition (i.e. the scale of competition must be greater than the scale of cooperation, [9,11]). In contrast, asocial individuals can benefit from smaller scale dispersal [25] to find patches with better resources or cooperative individuals to exploit without suffering the costs and risks of long distance dispersal. While *T. thermophila *shows this pattern, our current data does not allow us to evaluate whether the causes of dispersal differ between social and asocial strains (kin competition vs. resource availability respectively). However, a gradient of cooperative and dispersive strategies is an essential feature needed in an organism to realistically test the evolution of condition-dependent dispersal patterns.

## Conclusion

Advanced forms of social behavior benefit from stable population structure, so we expect a trade-off between social aggregation and dispersal and indeed find such patterns across species. Our results suggest such a dispersal-aggregation trade-off may exist among different cell lines within a species of single celled ciliates, *Tetrahymena thermophila*. However, the relationship between dispersal and cooperative aggregation may not be due to genetic effects and is much weaker than expected by theory given the impact of reduced colonization success suffered by aggregators. Our evidence suggests that the strength of the dispersal-aggregation trade-off is reduced by a number of specific adaptations including kin-recognition and a dispersal polymorphism that allow aggregators to disperse without losing all the benefits of aggregation. Such life history adaptations are likely a more common resolution to tension between dispersal and social aggregation than generally appreciated.

## Authors' contributions

The work presented here was carried out in collaboration between all authors. NS, EJF and JC defined the research theme. NS and EJF designed methods and experiments. EJF carried out the laboratory experiments. NS and AC analyzed the data and interpreted the results. All authors have contributed to the writing of the paper, and approved its final version.
